# EGCG promotes PRKCA expression to alleviate LPS-induced acute lung injury and inflammatory response

**DOI:** 10.1038/s41598-021-90398-x

**Published:** 2021-05-26

**Authors:** Mian Wang, Hua Zhong, Xian Zhang, Xin Huang, Jing Wang, Zihao Li, Mengshi Chen, Zhenghui Xiao

**Affiliations:** 1 Department of Epidemiology and Health Statistics, Xiangya School of Public Health, Central South University, Xiangya RD 110, Changsha, 410078 China; 2 Department of Cardiology, Xiangya Hospital, Central South University, Changsha, 410008 China; 3 Department of Epidemiology and Health Statistics, Hunan Normal University, Changsha, 410006 China; 4 Hunan Provincial Key Laboratory of Clinical Epidemiology, Central South University, Changsha, 410078 China; 5 Hunan Provincial Key Laboratory of Pediatric Emergency, Hunan Children’s Hospital, Changsha, 410006 China; 6 Department of Epidemiology and Health Statistics, School of Public Health, University of South China, Hengyang, 421001 China

**Keywords:** Biochemistry, Chemical biology, Diseases

## Abstract

Acute lung injury (ALI), which could be induced by multiple factors such as lipopolysaccharide (LPS), refer to clinical symptoms of acute respiratory failure, commonly with high morbidity and mortality. Reportedly, active ingredients from green tea have anti-inflammatory and anticancer properties, including epigallocatechin-3-gallate (EGCG). In the present study, protein kinase C alpha (PRKCA) is involved in EGCG protection against LPS-induced inflammation and ALI. EGCG treatment attenuated LPS-stimulated ALI in mice as manifested as improved lung injury scores, decreased total cell amounts, neutrophil amounts and macrophage amounts, inhibited the activity of MPO, decreased wet-to-dry weight ratio of lung tissues, and inhibited release of inflammatory cytokines TNF-α, IL-1β, and IL-6. PRKCA mRNA and protein expression showed to be dramatically decreased by LPS treatment while reversed by EGCG treatment. Within LPS-stimulated ALI mice, PRKCA silencing further aggravated, while PRKCA overexpression attenuated LPS-stimulated inflammation and ALI through MAPK signaling pathway. PRKCA silencing attenuated EGCG protection. Within LPS-induced RAW 264.7 macrophages, EGCG could induce PRKCA expression. Single EGCG treatment or Lv-PRKCA infection attenuated LPS-induced increases in inflammatory factors; PRKCA silencing could reverse the suppressive effects of EGCG upon LPS-stimulated inflammatory factor release. In conclusion, EGCG pretreatment inhibits LPS-induced ALI in mice. The protective mechanism might be associated with the inhibitory effects of PRKCA on proinflammatory cytokine release via macrophages and MAPK signaling pathway.

## Introduction

Acute lung injury (ALI) and the acute respiratory distress syndrome (ARDS) refer to clinical symptoms of acute respiratory failure, commonly with high morbidity and mortality^1^. ALI could be induced by severe sepsis, severe bacterial pneumonia, trauma, burn and other events. The pathological and physiological mechanism of ALI is thought to be related to the uncontrolled pulmonary inflammation^2–4^. ALI is characterized by increased production of proinflammatory transmitters, inflammation cell infiltration, and alveolar epithelial cell apoptosis^5,6^. Thus, the control of aberrant inflammation would efficiently improve the prognosis^7^.

Lipopolysaccharides (LPS) locates in the outer membrane of Gram-negative bacteria, acting as a key pathogen in animal models of ALI^8^. Stimulation with LPS leads to increased expression of toll like receptor 4 (TLR4) and its binding to adaptor molecules, triggering the activation of NF-κB and the release of pro-inflammatory cytokines, including tumor necrosis factor-α (TNF-α), interleukin (IL)-1β, and IL-6, which cause inflammatory responses and lead to severe pulmonary damage^9,10^. Notable progress has been made in reducing ALI, however, the morbidity and mortality rates of human ALI remain stubbornly high. Hence, there is a pressing need for studying a new effective treatment for patients with ALI and possible ARDS.

Reportedly, green tea has anti-inflammatory and anticancer properties^11–13^. Green tea contains epigallocatechin-3-gallate (EGCG), (–)-epigallocatechin, (–)-epicatechin, (+)-gallocatechin, and many other polyphenols, among which the most abundant and the major bioactive constituent is EGCG^14–16^. As previously reported, EGCG contributes to repressing inflammatory responses^12^. Reportedly, EGCG inhibits the production of IL-6 and IL-8 within HMC-1 cells^17^, A549 bronchial epithelial cells^18,19^, HT29 cells^20,21^, and T84 cells^20^. In addition, EGCG was known to inhibit LPS-stimulated TNF-α production from the murine macrophage cell line, RAW 264.7^22^. Another report showed that EGCG decreased the expression levels of proinflammatory transmitters TNF-α, IL-1β, and IL-6 within the lung, serum, and bronchoalveolar lavage fluid (BALF) in LPS-stimulated ALI mice in vivo^23^. There is growing evidence that EGCG suppresses inflammatory responses to improve LPS-induced ALI; thus, investigating the mechanism underlying EGCG protection against LPS-induced ALI might extend its clinical application.

To identify factors that interact with EGCG to participate in EGCG protection against LPS-induced ALI, the present study searched PubChem website (https://pubchem.ncbi.nlm.nih.gov/) and selected ten candidate proteins with the highest interaction score for further validation. Among the proteins that could be significantly altered by LPS stimulation, PRKCA (protein kinase C alpha) belongs to protein kinase C (PKC) family and was previously reported to be involved in H_2_O_2_-induced injury to A549 cells^24^; H_2_O_2_ exposure decreased PKC-α activity^24^. Besides, the PKC family is involved in the regulation of inflammation. Yao et al*.* reported that protein kinase C zeta mediates cigarette smoke/aldehyde- and lipopolysaccharide-induced lung inflammation^25^. Hsu et al*.* identified that PRKCA involved in nanoparticles-induced inflammation and oxidative stress of lung epithelial A549 cells^26^. Therefore, we hypothesized that PRKCA could also contribute to EGCG protection against LPS-stimulated inflammation and ALI.

Herein, the study established LPS-stimulated ALI model within BALB/c mice, treated or non-treated with EGCG, and examined the changes in pathological characteristics, lung injury scores, cell numbers, the activity of myeloperoxidase (MPO), wet-to-dry weight ratio of lung tissues and inflammation cytokine levels within BALF to examine LPS-stimulated ALI and the protective effects of EGCG. PRKCA mRNA and protein expression showed to be determined in LPS-stimulated ALI mice in the presence or absence of EGCG treatment. Next, PRKCA silencing or overexpression was achieved in LPS-induced ALI mice by infecting lentivirus (Lv-shPRKCA or Lv-PRKCA) and examined the related indexes in the presence or absence of EGCG treatment. Finally, LPS-induced mouse macrophage RAW 264.7 cells showed to be infected with Lv-shPRKCA or Lv-PRKCA and examined for the protein levels of PRKCA and inflammatory cytokines.

## Materials and methods

### Animal

Male BALB/c mice (pathogen free; 16–20 g of weight) were obtained from Hunan SJA Laboratory Animal Co., Ltd (Changsha, China) and raised in a pathogen free condition. The mice were kept in temperature-controlled rooms in a 12:12 h light–dark cycle and provided with food and water at will. Before the study, the animals underwent at least seven days of adaptation. All procedures involving animals have been approved by the Animal Experiment Ethics Committee of Central South University. All procedures were conducted based on recommendations in guidelines for the care and use of laboratory animals. All procedures were performed using the 30 mg/kg doses of 2% pentobarbital sodium by intraperitoneal injection anesthesia and every effort was made to minimize the pain for mice.

### LPS-induced acute lung injury mouse model

A total of 40 male BALB/c mice were randomly divided into 4 groups: the control group in which mice only received sterile saline treatment, the EGCG group in which mice only received EGCG treatment, the LPS group in which mice only received LPS stimulation, and the LPS + EGCG group in which mice received LPS stimulation and EGCG treatment. For LPS stimulation, each mouse was anesthetized with pentobarbital sodium by intraperitoneal injection and intratracheally injected by LPS (5 mg/Kg b.wt)^27^. The mice were then placed vertically and rotated for 1 min to distribute the drops to the lungs. For EGCG treatment, mice were administered EGCG (10 mg/kg b.wt, intraperitoneal injection) 1 h prior to LPS injection. The administration and dose of EGCG were selected according to a previous study^28^. Mice were euthanized to collect study samples at 24 h after the intratracheal administration of LPS.

For PRKCA silencing or overexpression, each mouse was injected through the caudal vein with 2 × 10^7^ transducing units (TU) of Lv-shPRKCA or Lv-PRKCA (Lv-NC used as a negative control; Genetop, Changsha, China) lentivirus. After 5 days, mice were treated or non-treated with EGCG and intratracheally injected with LPS as described above.

### Bronchoalveolar lavage fluid (BALF) and cells counting

After anesthetizing and sacrificing the mice, the lungs of the mice were lavaged with 1.0 mL PBS (pH 7.2) 3 times, the upper trachea was intubated, and the BALF of the mice was collected. The BALF was centrifuged at 700 × *g* for 5 min below 4 °C. The pelleted cells were resuspended in 50 μL of PBS and stained with Diff-Quik (International Reagents Corp., Kobe, Tokyo, Japan) to prepare for cell counting. Then, cell numbers were double-blind counted, including total cells, neutrophils, macrophages and lymphocytes with a hemocytometer and Giemsa staining^29,30^.

### Histopathologic examination and lung injury evaluation

Lung tissues were collected and fixed in 10% formalin, embedded in paraffin, sliced into 5-μm thick sections, and stained with hematoxylin and eosin (H&E) for the detection of the pathological changes in the lung tissues. Finally, histopathology was observed under an optical microscope (DSX100, Olympus, Tokyo, Japan). Lung injury score was measured according to the methods reported previously^31,32^. The criteria are as follows: score 0 = no damage, score l = mild damage, score 2 = moderate damage, score 3 = severe damage, score 4 = very severe histologic changes.

### ELISA analysis

BALF levels of inflammatory factors, including TNF-α, IL-6, and IL-1β were quantified using specific ELISA kits, Mouse TNF-α ELISA Kit (ab208348, Abcam, Cambridge, MA, USA), Mouse IL-6 ELISA Kit (ab100712, Abcam), IL-1β (Mouse) ELISA Kit (SK00746-03; AVISCERA BIOSCIENCE, Santa Clara, CA, USA) for mouse following the protocols.

### MPO activity assay

After BALF collection, the upper left leaf was collected for MPO activity (units of MPO activity/g of lung tissue weight) examination in a H_2_O_2_/o-dianisidine buffer using a spectrophotometer (Shanghai Precision & Scientific Instrument Co. Ltd, China) at 460 nm.

### Lung weight ratio (wet/dry)

The severity of pulmonary edema was evaluated by calculating the wet/dry ratio (W/D ratio). The right lower lungs of mice in each group were weighed and then dehydrated at 60 °C for 72 h in an oven.

### Cell line

Mouse macrophage cell line, RAW 264.7, was purchased from ATCC (TIB-71, Manassas, VA, USA) and cultured in Dulbecco's Modified Eagle's Medium (Catalog No. 30-2002, ATCC) supplemented with 10% fetal bovine serum (FBS) (Invitrogen, Carlsbad, CA, USA).

PRKCA silencing was achieved by infecting cells with lentivirus containing small hairpin RNA targeting PRKCA (Lv-shPRKCA; Lv-NC used as a negative control; Genetop). PRKCA overexpression was achieved by infecting cells with lentivirus overexpressing PRKCA (Lv-PRKCA; Lv-NC used as a negative control; Genetop).

For LPS stimulation, cells were activated by incubation in a medium containing LPS (100 ng/ml) when the cells grew to a density of 2 × 10^6^ cells/ml.

### Isolation of alveolar macrophages

The isolation and characterization of murine alveolar macrophages were perform based on previous research^33,34^. In brief, BALF pellet from all mice groups was seeded in Petri dishes with RPMI 1640 Medium supplemented with 10% FBS, 100 IU/mL penicillin and 100 µg/mL streptomycin (Gibco BRL, Grand Island, USA), for 1 h at 37 °C. The supernatant was discarded and purified attached alveolar macrophages were cryopreserved in 500 µL of TRIzol reagent (Sigma-Aldrich, St. Louis, USA). Alveolar macrophages' purity was assessed by Diff-Quick staining and immunofluorescence.

### Polymerase chain reaction (PCR)-related analysis

Total RNA was isolated from tissue samples by Trizol reagent (Invitrogen). The PCR-related analyses were performed using a reaction system described before^35^ for 35 cycles. The mouse GAPDH housekeeping gene was used as an internal control. The relative expression levels were calculated using the 2^−ΔΔCT^ method.

### Immunoblotting

The protein levels of TNF-α, IL-6, IL-1β, p-p38, p38, p-ERK1/2, ERK1/2, p-JNK, JNK and PRKCA were determined by Immunoblotting. Protein samples were collected from target tissues or cells. After examining for the protein concentrations using a bicinchoninic acid assay kit (Pierce, Rockford, IL, USA), proteins were separated in 10% Tris-glycine SDS polyacrylamide gel and the protein bands were then transferred to immobile polyvinylidene fluoride (PVDF) membranes. The membranes were incubated in blocking solution for 1 h at room temperature for blocking unspecific bindings, and then incubated with proper antibodies against TNF-α (ab8348; Abcam, Cambridge, MA, USA), IL-6 (66146-1-Ig; Proteintech, Rosemont, IL, USA), IL-1β (ab9722, Abcam), p-p38 (ab4822, Abcam), p38 (ab31828, Abcam), p-ERK1/2 (ab214362, Abcam), ERK1/2 (ab184699, Abcam), p-JNK (ab124956, Abcam), JNK (ab208035, Abcam) or PRKCA (MBS9133385; MyBioSource, San Diego, CA, USA) at 4 °C overnight. Then, the membranes were incubated with the proper secondary antibody at room temperature for 1 h. Signals were visualized using enhanced chemiluminescence (ECL) agent and the relative protein levels were calculated normalizing to GAPDH.

### Statistics analysis

Results were collected from at least three independent experiments. All the data are processed with GraphPad (San Diego, CA, USA) and shown as mean ± standard deviation (S.D.). Statistical significance was evaluated by one-way analysis of variance (ANOVA) followed by Tukey's multiple comparison test or by Student's *t*-test. A *P* value of < 0.05 is considered statistically significant.

### Ethics approval and consent to participate

All procedures performed in studies involving animals were in accordance with the ethical standards of Central South University and with the 1975 Helsinki declaration and ARRIVE guidelines.

## Results

### EGCG alleviates LPS-stimulated ALI within mouse model

**Figure 1:**
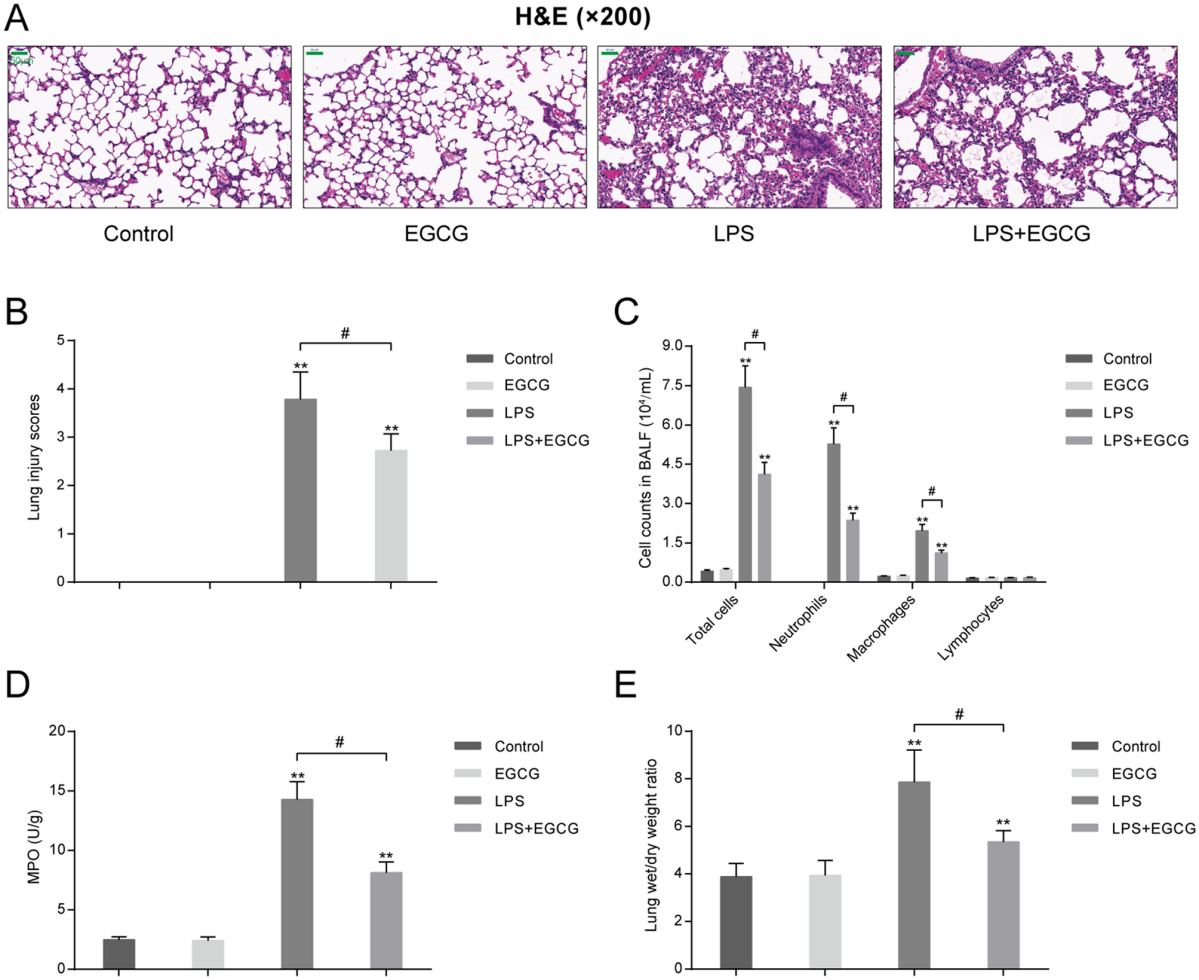
Epigallocatechin gallate (EGCG) alleviates LPS-induced acute lung injury (ALI) in mouse model. Mice were randomly divided into four groups: the control group, the EGCG group, the LPS group, and the LPS + EGCG group. Mice in each group were treated accordingly and examined for (**A**) the pathological changes in lung tissues by hematoxylin and eosin (H&E) staining (200 ×). scale bar = 50 μm; (**B**) lung injury scores following the methods reported before; (**C**) bronchoalveolar lavage fluid (BALF) cell numbers including total cells, neutrophils, macrophages and lymphocytes using a hemocytometer and Giemsa staining; (**D**) MPO activity using a spectrophotometer; (**E**) lung wet/dry weight ration. **P < 0.01 compared to Control group; #*P* < 0.05 compared to LPS group.

To confirm the specific roles of EGCG in LPS-induced ALI, the study established the murine model of LPS-stimulated ALI. We randomly divided the mice into four groups: the control group, the EGCG group, the LPS group, and the LPS + EGCG group, which received corresponding treatment. H&E staining on mice lung showed that, in comparison with the control and single EGCG group, single LPS treatment induced lung injury, as manifested as inflammatory cell infiltration, edema and hemorrhage and subdued necrosis, whereas EGCG treatment on LPS-stimulated mice alleviated LPS-induced lung injury (Fig. 1A). The results of H&E staining were further evidenced by lung injury score evaluation that LPS stimulation dramatically increased the lung injury score, which was observably decreased by about 30% by EGCG co-treatment when compared to LPS group (*P* < 0.05, Fig. 1B). Consistently, LPS stimulation induced sharp increases in total cell number and neutrophil number and a significant increase in macrophage number, whereas caused no change in lymphocyte number when compared to control group (*P* < 0.01, Fig. 1C); LPS-induced increases in total cell, neutrophil, and macrophage numbers were significantly attenuated by EGCG co-treatment (*P* < 0.05, Fig. 1C). Moreover, LPS stimulation significantly enhanced MPO activity (*P* < 0.01, Fig. 1D) and increased lung wet/dry weight ratio (*P* < 0.01, Fig. 1E), which could both be reduced by about 40% and 30% respectively by EGCG co-treatment when compared to LPS group (*P* < 0.05, Fig. 1D,E). These data suggest that LPS induces ALI in mice lung, while EGCG treatment attenuates ALI in mice induced by LPS.

### EGCG decreases the levels of the inflammatory cytokines in vivo

**Figure 2:**
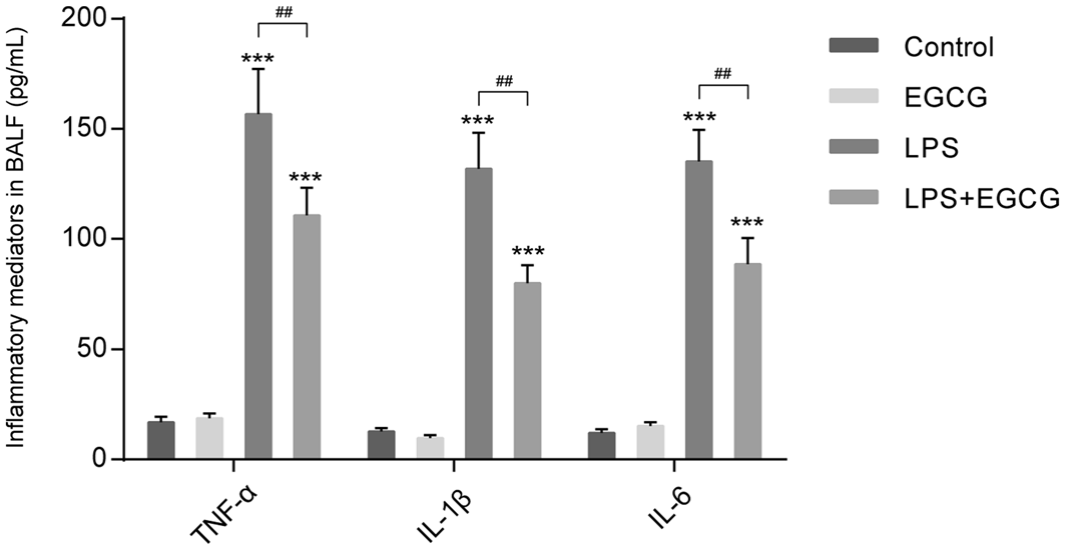
EGCG decreases the levels of the inflammatory cytokines in vivo. The BALF levels of inflammatory factors, including TNF-α, IL-6, and IL-1β in each group were examined by ELISA using corresponding mouse ELISA kits. ***P < 0.001 compared to Control group; ##*P* < 0.001 compared to LPS group.

To further validate the inflammation in mice lung in each group, the expression levels of inflammatory factors, including TNF-α, IL-6, IL-1β, in BALF were examined. Figure 2 showed that when compared with control group, LPS treatment dramatically increased TNF-α, IL-6, and IL-1β levels (*P* < 0.001); while compared to LPS group, EGCG decreased the levels of TNF-α, IL-6, and IL-1β by about 30%, 40% and 35% respectively (*P* < 0.01). In summary, LPS stimulation induces inflammation reaction within mice lung, which could be attenuated by EGCG treatment.

### EGCG modulates PRKCA expression within LPS-mediated ALI mice

To further investigate the mechanism of EGCG improving LPS-induce ALI, we screened for proteins that interact with EGCG and proteins with top ten interaction scores were shown in Table 1.

**Table 1:** Proteins predicted to interact with EGCG according to PubChem.

COMPOUND	PREDICTED TARGETS [GENE SYMBOL] RANKED ACCORDING TO THE DECREASING (SCORE)
**PUBCHEM_CID:65,064**	SEC14L3(114.143)PPP2CA(114.143)PRKCA(114.143)NR1I2(114.143)ALOX5(114.143) PPP2CB(114.143)SEC14L2(114.143)DGKA(114.143)PRKCB(114.143)SEC14L4(114.143) SOAT1(48.000)MTTP(48.000)SOAT2(48.000)SEC14L6(25.857)

**Figure 3:**
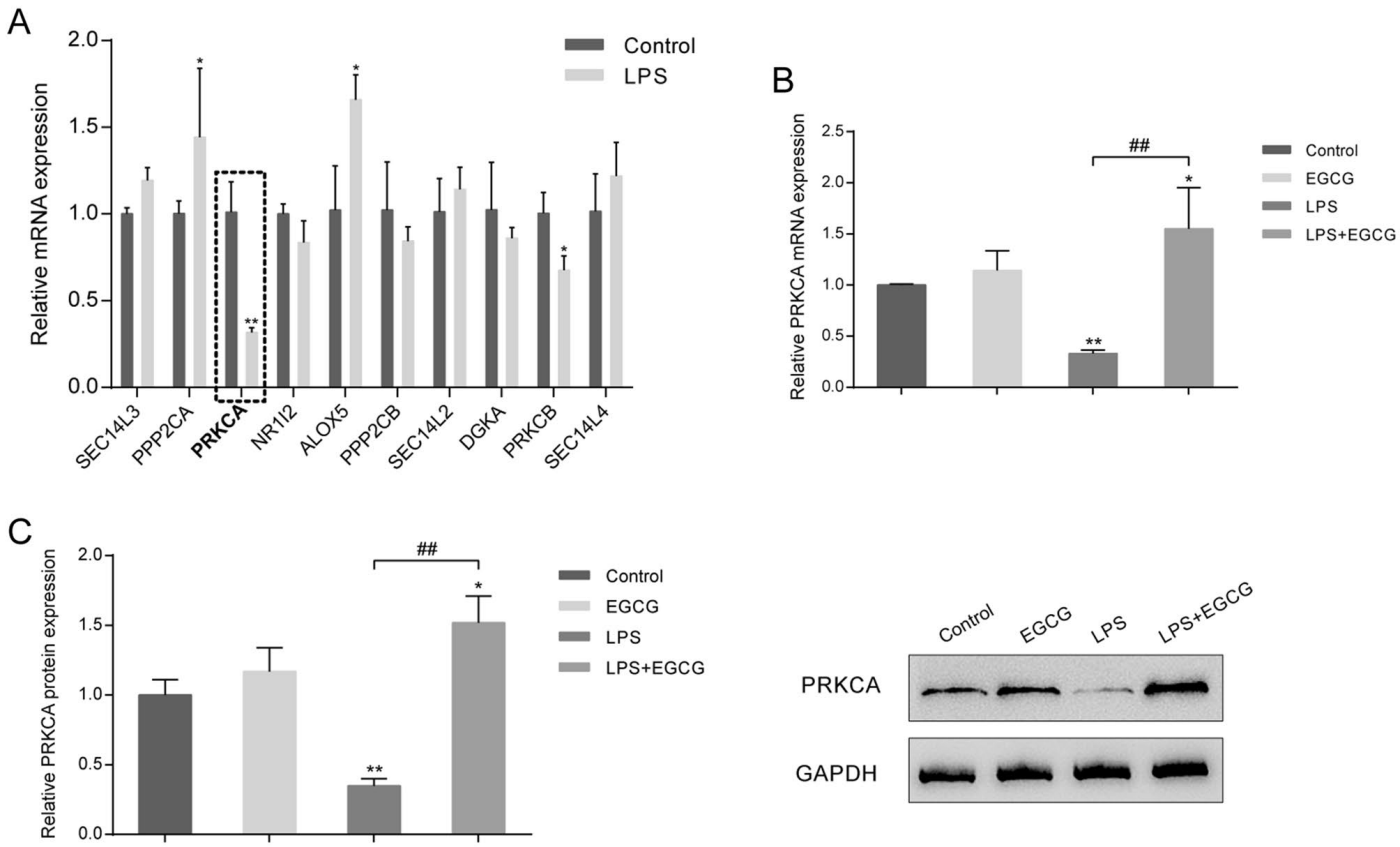
EGCG modulates PRKCA expression in LPS-induced ALI mice. Mice were divided into four groups (the control group, the EGCG group, the LPS group, and the LPS + EGCG group) accordingly. (**A**) The expression levels of the top ten genes predicted to interact with EGCG were examined in the control group and the LPS group using real-time qPCR. (**B**) PRKCA mRNA expression in mouse lung tissues from each group was determined using real-time qPCR. (**C**) PRKCA protein levels in mouse lung tissues from each group were determined using Immunoblotting. *P < 0.05, **P < 0.01 compared to Control group; ##*P* < 0.01 compared to LPS group.

Mice were divided into four groups as mentioned above and the expression of the top ten genes predicted to interact with EGCG showed to be examined within the control and the LPS group. As shown in Fig. 3A, compare to control group, LPS stimulation significantly inhibited PRKCA and PRKCB expression by about 70% and 30%, respectively (*P* < 0.05). But, as displayed in Fig. S1, LPS stimulation observably restrained PRKCB mRNA (*P* < 0.05, Fig. S1A) and protein (*P* < 0.05, Fig. S1B) expression; while EGCG had no significant effect on the mRNA and protein expression of PRKCB. And, in mice lung, LPS stimulation significantly reduced by about 65% when compared with control group; while EGCG treatment reversed the mRNA expression (*P* < 0.01, Fig. 3B) and the protein levels (*P* < 0.01, Fig. 3C) of PRKCA, in comparison with the control group and single LPS group. Moreover, PRKCA might be involved in H_2_O_2_-induced injury to A549 cells^24^; H_2_O_2_ exposure decreased protein kinase C (PKC)-α activity^24^. Thus, we chose PRKCA for further experiments. These data further suggest that PRKCA might participate in the protective effects of EGCG against LPS-mediated ALI in mice.

### PRKCA effects upon LPS-mediated ALI

**Figure 4:**
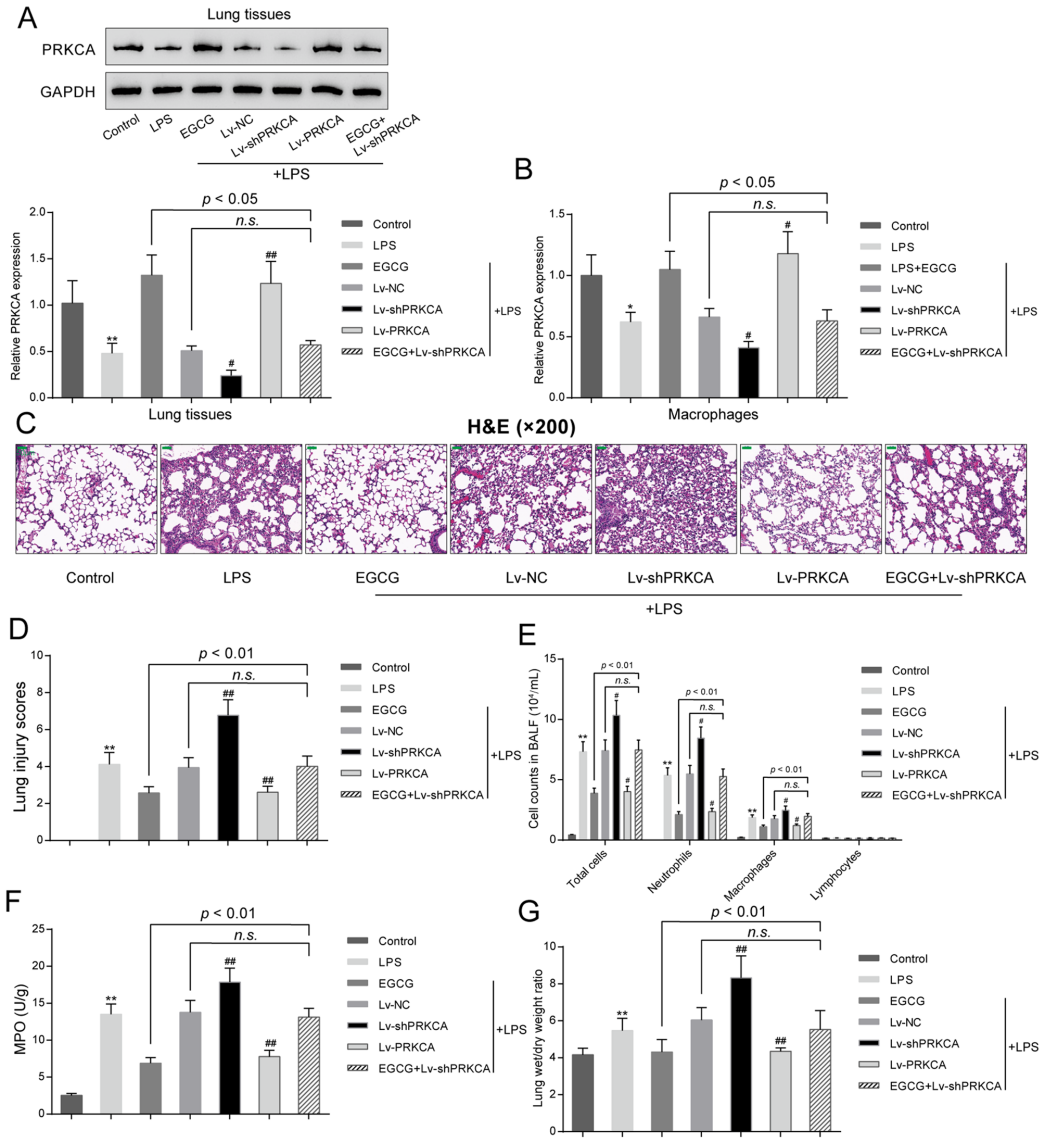
Effects of PRKCA on LPS-induced ALI. Mice were randomly divided into seven groups: the control group, the LPS group, the LPS + EGCG, the LPS + Lv-NC group, the LPS + Lv-shPRKCA group, the LPS + Lv-PRKCA group, and the LPS + EGCG + Lv-shPRKCA group. Mice in each group received treatment and/or injection accordingly. (**A**) PRKCA mRNA and protein expression levels in lung tissues of mice from each group was determined using real-time qPCR and Immunoblotting; (**B**) PRKCA expression levels in alveolar macrophages of mice lung from each group were determined using real-time qPCR; (**C**) pathological changes using H&E staining (200 ×). scale bar = 50 μm; (**D**) lung injury scores following the methods reported before; (**E**) cell numbers including total cells, neutrophils, macrophages and lymphocytes using a hemocytometer; (**F**) MPO activity using a spectrophotometer; (**G**) lung wet/dry weight ration. *P < 0.05, **P < 0.01, compared to the control group. #*P* < 0.05, ##*P* < 0.01, compared to the LPS + Lv-NC group.

To validate the specific roles of PRKCA in EGCG protection against LPS-mediated ALI, we randomly divided the mice into seven groups: the control group, the LPS group, the LPS + EGCG group, the LPS + Lv-NC group, the LPS + Lv-shPRKCA group, the LPS + Lv-PRKCA group, and the LPS + EGCG + Lv-shPRKCA group, which received treatment and/or injection accordingly. In lung tissues of mice from the LPS, LPS + Lv-NC, and LPS + Lv-shPRKCA groups, PRKCA mRNA and protein expression showed to be dramatically decreased than those in the control group (*P* < 0.01, Fig. 4A). Upon LPS stimulation, EGCG treatment and Lv-PRKCA infection significantly increased PRKCA mRNA expression and protein levels, and EGCG treatment also relieved the effect of PRKCA silencing on PRKCA mRNA expression and protein levels (Fig. 4A). In macrophages of alveolar, when compared to control group, PRKCA expression level was markedly decreased in the LPS group (*P* < 0.05, Fig. 4B). Upon LPS stimulation, EGCG treatment and Lv-PRKCA infection notably increased PRKCA expression level; knockdown of PRKCA further inhibited PRKCA expression level; and EGCG treatment reversed the inhibitory effect of PRKCA silencing on PRKCA expression level (*P* < 0.05, Fig. 4B). As for lung injury evaluation, H&E staining and lung injury scores indicated that PRKCA silencing further aggravated LPS-mediated pulmonary injury (Fig. 4C,D), EGCG treatment and PRCKA overexpression improved LPS-mediated pulmonary injury (Fig. 4C,D), and EGCG treatment-induced improvement of LPS-induced lung injury was significantly abolished by PRKCA silencing (Fig. 4C,D). Moreover, LPS-induced increases in total cell amounts, neutrophil amounts, and macrophage amounts were further increased by PRKCA silencing while decreased by EGCG treatment and PRKCA overexpression; PRKCA silencing attenuated EGCG effects upon LPS-mediated increases in cell numbers (Fig. 4E). Moreover, the activity of MPO and the wet-to-dry weight ratio of lung issues showed the similar trend, that is, LPS significantly increased, whereas PRKCA silencing further increased the activity of MPO and the wet/dry ratio; LPS-induced increases were reduced via EGCG treatment and PRKCA overexpression (Fig. 4F,G). EGCG treatment attenuated LPS-mediated elevations in these indexes, while PRKCA silencing attenuated the effects of EGCG treatment (Fig. 4F,G). These data suggest that PRKCA silencing further aggravates LPS-induced lung injury and attenuates EGCG protection against LPS-mediated pulmonary injury.

### PRKCA effects upon the levels of the inflammatory cytokines in vivo through MAPK signaling pathway

**Figure 5:**
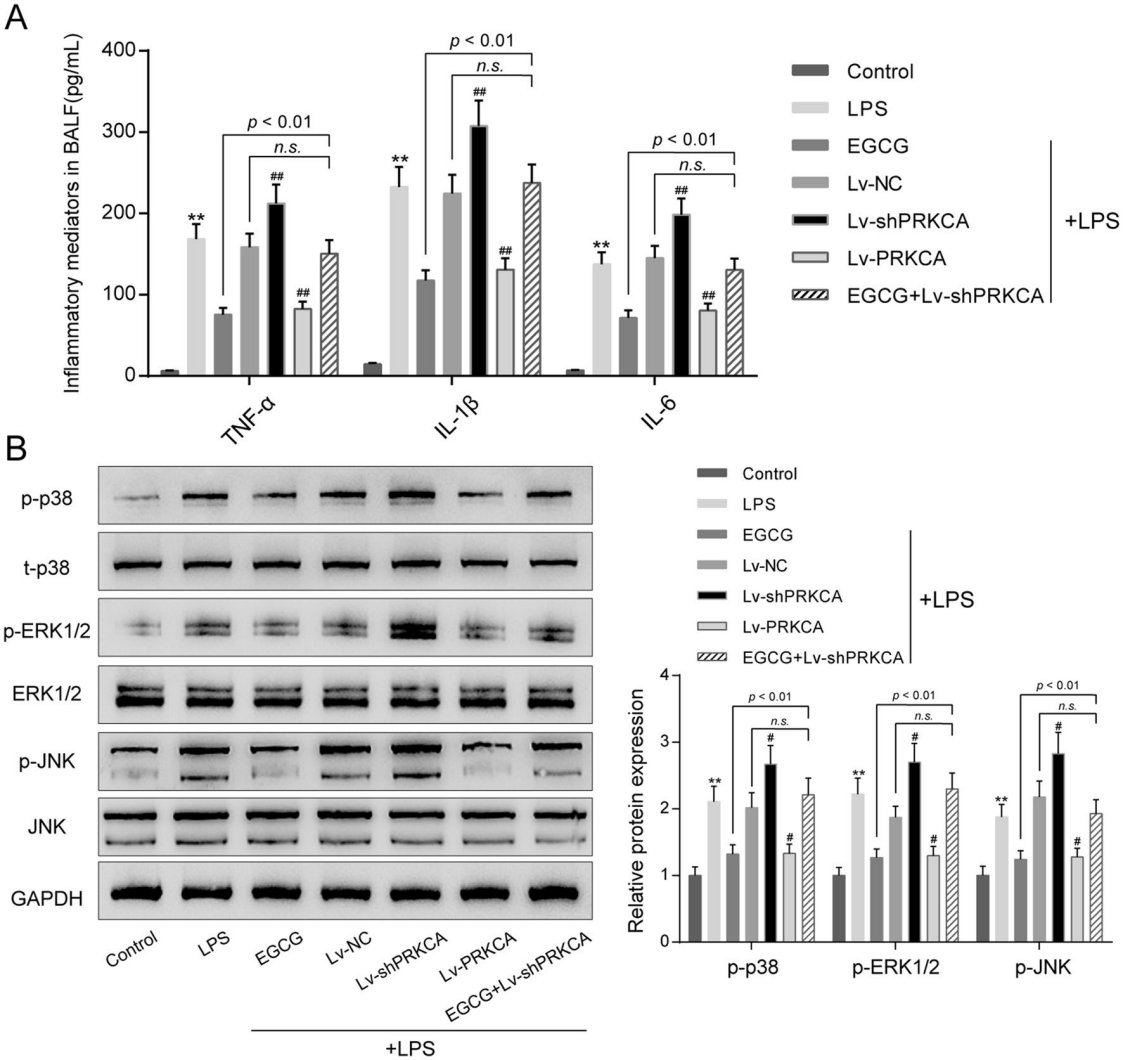
Effects of PRKCA on the levels of the inflammatory cytokines in vivo through MAPK signaling pathway. Mice were grouped and treated accordingly. (**A**) The bronchoalveolar lavage fluid (BALF) levels of inflammatory factors, including TNF-α, IL-6, and IL-1β in each group were examined by ELISA using corresponding mouse ELISA kits. (**B**) The protein expression levels of MAPK signaling pathway-related proteins, including p-p38, p38, p-ERK1/2, ERK1/2, p-JNK and JNK in each group were examined by Immunoblotting. **P < 0.01, compared to the control group. #*P* < 0.05, compared to the LPS + Lv-NC group.

Regarding the inflammatory microenvironment in LPS-mediated ALI mice, we examined the BALF levels of inflammatory factors (TNF-α, IL-6, and IL-1β) within each group. LPS stimulation significantly enhanced TNF-α, IL-6, and IL-1β levels (*P* < 0.01); PRKCA silencing further increased, while EGCG treatment and PRKCA overexpression decreased LPS-mediated increases within the levels of TNF-α, IL-6, and IL-1β (Fig. 5A). EGCG reduced TNF-α, IL-6, and IL-1β levels upon LPS stimulation, and EGCG effects were significantly reversed by PRKCA silencing (*P* < 0.01, Fig. 5A).

According to the KEGG website (https://www.kegg.jp/kegg-bin/show_pathway?ko04010+K02677)^36^, protein kinase C (PKC) family is located upstream of MAPK signaling pathway, affecting a variety of physiological processes through the MAPK signaling pathway. Moreover, previous studies had shown that MAPK signaling pathway is involved in the process of lung injury^37–39^. Hence, we hypothesized that PRKCA alleviated acute lung injury in mice by regulating MAPK signaling pathway. Then, we detected the expression levels of MAPK signaling pathway-related proteins (p-p38, p38, p-ERK1/2, ERK1/2, p-JNK, JNK) in each group (Fig. 5B). LPS stimulation observably enhanced p-p38, p-ERK1/2, and p-JNK protein levels about 210%, 220% and 180% respectively when compared with control group (*P* < 0.01); knockdown of PRKCA further increased, while EGCG treatment and overexpression of PRKCA notably decreased LPS-mediated increases within p-p38, p-ERK1/2, and p-JNK protein levels. However, when compared with LPS + Lv-NC group, the inhibitory effect of EGCG on p-p38, p-ERK1/2, and p-JNK protein levels couldn't observed in LPS + EGCG + Lv-shPRKCA group; the protective effect of EGCG might be eliminated by the effect of silencing of PRKCA (*P* < 0.01, Fig. 5B). In summary, PRKCA might participate in the protective effects of EGCG against LPS-stimulated pulmonary injury through modulating inflammatory factor release and MAPK signaling pathway.

### Effects of PRKCA on LPS-induced inflammatory responses through MAPK pathway in vitro

**Figure 6:**
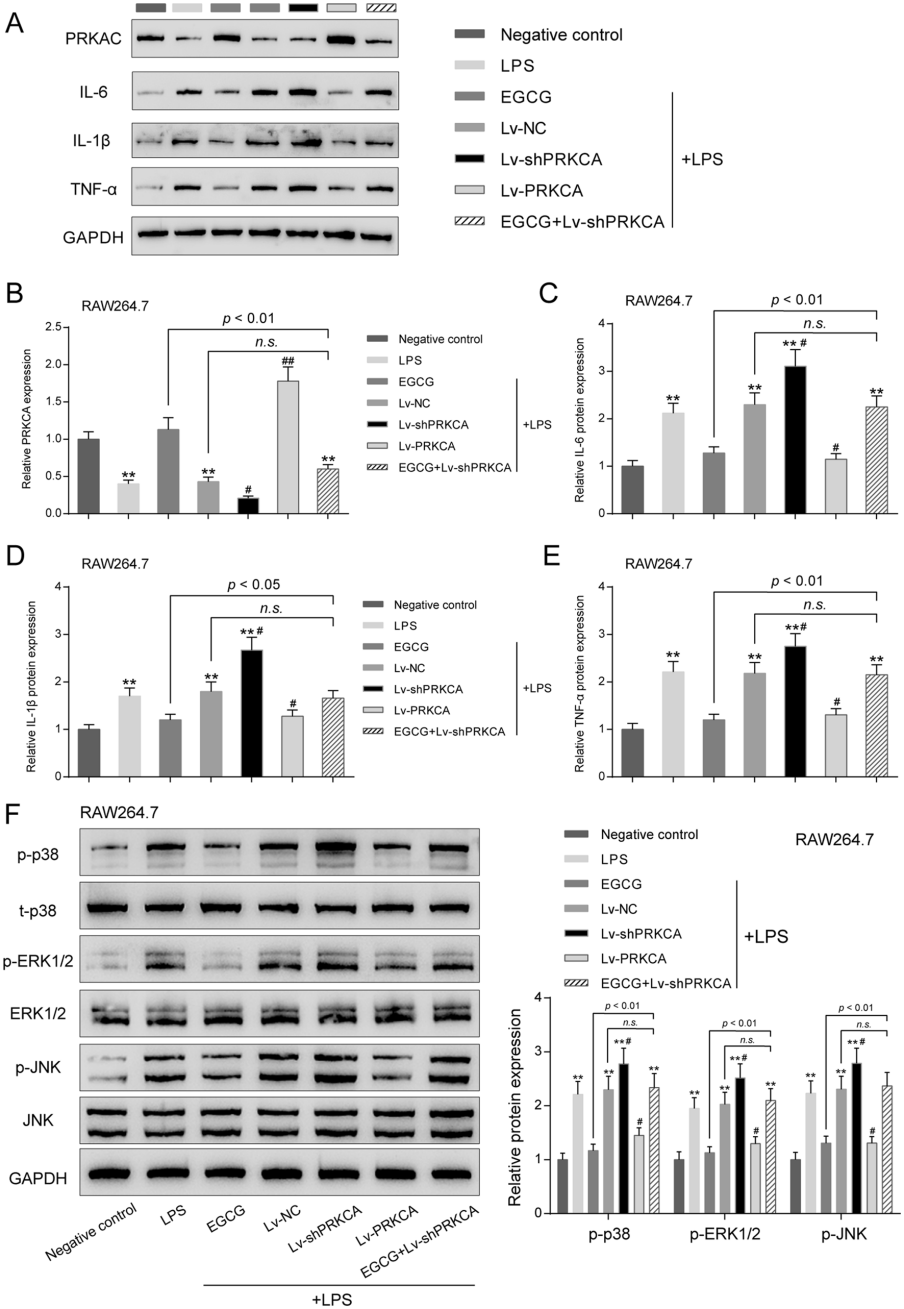
Effects of PRKCA on LPS-induced inflammatory responses through MAPK pathway in vitro. Mouse macrophage RAW 264.7 cells were divided into seven groups: the negative control group, the LPS group, the LPS + EGCG group, the LPS + Lv-NC group, the LPS + Lv-shPRKCA group, the LPS + Lv-PRKCA group, and the LPS + EGCG + Lv-shPRKCA group. Cells in each group were treated and/or infected accordingly. (**A**–**E**) The protein levels of PRKCA, IL-6, IL-1β, and TNF-α were determined using Immunoblotting. (**F**) The protein expression levels of MAPK signaling pathway-related proteins, including p-p38, p38, p-ERK1/2, ERK1/2, p-JNK and JNK in each group were detected using Immunoblotting. **P < 0.01 compared to the control group; #*P* < 0.05, ##*P* < 0.01 compared to the LPS + Lv-NC group.

To investigate the specific role of PRKCA in inflammatory factor release, the study treated mouse macrophage RAW 264.7 cells in different ways. We divided RAW 264.7 cells into seven groups: the negative control group, the LPS group, the LPS + EGCG group, the LPS + Lv-NC group, the LPS + Lv-shPRKCA group, the LPS + Lv-PRKCA group, and the LPS + EGCG + Lv-shPRKCA group. Cells in each group were treated and/or infected accordingly. As observed in tissue samples, PRKCA protein level was significantly decreased by about 60% by LPS stimulation compared to control group (*P* < 0.01, Fig. 6A,B). Under LPS stimulation, when compared with LPS group, EGCG or Lv-PRKCA infection restored, while Lv-shPRKCA further decreased PRKCA protein level; the effects of EGCG on PRKCA protein level were reversed by Lv-shPRKCA infection (Fig. 6A,B). These data indicate that EGCG could upregulate PRKCA expression in mouse macrophages under LPS stimulation.

As for inflammatory factors, LPS promoted IL-6, IL-1β, and TNF-α protein contents. Single EGCG treatment or Lv-PRKCA infection attenuated LPS-induced increases in inflammatory factors (Fig. 6A,C–E), while Lv-shPRKCA infection further increased LPS-induced increases in these factors (Fig. 6A,C–E). The effects of EGCG on inflammatory factors were partially reversed by Lv-shPRKCA infection (Fig. 6A,C–E).

As for MAPK signaling pathway-related proteins (p-p38, p38, p-ERK1/2, ERK1/2, p-JNK, JNK), LPS notably facilitated p-p38, p-ERK1/2, and p-JNK protein contents. Single EGCG treatment or Lv-PRKCA infection relieved LPS-induced increases in MAPK signaling pathway-related proteins, while overexpression of PRKCA further promoted LPS-induced increases in these proteins. The inhibitory effects of EGCG on MAPK signaling pathway-related proteins were partially reversed by knockdown of PRKCA (Fig. 6F). These data suggest that PRKCA silencing could reverse the inhibitory effects of EGCG on inflammatory factor release through regulating MAPK signaling pathway.

## Discussion

Herein, this study revealed that PRKCA is involved in EGCG protection against LPS-induced inflammation and ALI. CALI is a frequent complication following sepsis in critically ill patients and LPS is thought to be the most important pathogen that leads to the development of ALI in sepsis^40^. As previously reported, LPS-stimulated ALI is related to elevated infiltration of inflammatory cells and significant pulmonary damage, with the major characteristic features of the alterations within histopathological characteristics and other markers^40,41^. In the present study, both H&E staining and lung injury scoring showed that LPS stimulation caused significantly lung injury to mice lung, whereas EGCG treatment improved LPS-induced histopathological changes and lung injury.

The pathological process of LPS-stimulated ALI involves the TLR4/NF-κB pathway, which initiates intracellular inflammatory signal transduction^42,43^. Following TLR4 treatment and LPS treatment, MyD88 pathway can transport signals to TNF receptor-associated factor-6 (TRAF6), resulting in IκB degradation and NF-κB activation^42,43^. The nucleus-translocation of NF-κB results in increases in proinflammatory transmitters^44^, thereby amplifying a cascade of inflammation, promoting the transfer of neutrophils into alveoli and damaging pulmonary tissues^42,43^. Myeloperoxidase (MPO) activity was applied to assess the activation and accumulation of neutrophils and polymorphonuclear leukocyte in the lung tissues^45,46^. Herein, LPS indeed elevated the total cell number, the neutrophil numbers, and the macrophage number in the BALF, and the MPO activity, while EGCG treatment significantly attenuated these changes induced by LPS, indicating that EGCG treatment alleviates LPS-triggered inflammatory cascade.

Gram-negative bacterial infections are the major cause of ALI^28^. The recognition of LPS that has entered the body is achieved through specialized receptors known as pattern recognition receptors (PPRs), which plays a role in inflammatory signal transduction, leading to the synthesis and release of pro-inflammatory cytokines, such as TNF-α, IL-1β, and IL-6. The imbalance between pro-inflammatory and anti-inflammatory transmitters is a vital event during ALI development^9,10^. The macrophages and endothelial cells stimulated with TNF-α produce substantial amounts of proinflammatory transmitters, including IL-1β and IL-8, and secondary inflammatory mediators, including platelet activator and NO^47^. IL-1β contribute to promoting tissue cell sensitivity to TNF-α and stimulating other inflammatory cytokines, including TNF-α, IL-8, E-selectin, and P-selectin. IL-6 induces acute inflammation, enhances neutrophil recruitment and activation, leads to lung edema, and causes systemic inflammation reaction^9,47^. These three pro-inflammatory cytokines of TNF-α, IL-1β, and IL-6 enhance each other and trigger a cascade of inflammation response resulting in ALI. In the present study, EGCG significantly reduced LPS-mediated elevations within the levels of TNF-α, IL-1β, and IL-6, further confirming EGCG protection against LPS-mediated lung injury via improving the cascade of inflammation reaction.

As for the mechanism underlying the protective effects of EGCG, herein, we revealed that PRKCA expression, which is predicted to interact with EGCG, could be significantly downregulated by LPS stimulation. The PRKCA has been reported to mediate many physiological activities and the role of PRKCA in lung injury may be controversial. Liu et al*.* elucidated that the PKC signaling pathway were activated by mechanical ventilation in ventilator-induced lung injury^48^. The activation of protein kinase C participated in lung injury in endotoxemic sheep^49^. However, PRKCA may also plays a protective role in lung injury and impairs inflammatory response. The inflammatory responses in the airways are related to the generation of reactive oxygen species (ROS), including H_2_O_2_ and superoxide (O_2_^-^), leading to widespread pulmonary damage within respiratory illness; notably, H_2_O_2_ exposure decreased PRKCA activity by causing translocation of PRKCA from the membrane to the cytoplasm^24^. Moreover, Lin et al*.* illuminated that up-regulation of PYK2/PRKCA-dependent haem oxygenase-1 attenuates TNF-α-induced lung inflammation^50^. In the present study, LPS significantly downregulated, whereas EGCG treatment significantly reversed PRKCA mRNA expression and protein levels, suggesting that PRKCA might participate in EGCG protection against LPS-mediated pulmonary damage. As expected, PRKCA silencing further aggravated, whereas PRKCA overexpression attenuated LPS-mediated pulmonary damage via improving histopathological alterations, reducing cell numbers, inhibiting the activity of MPO, and reducing proinflammatory cytokine levels. More importantly, after silencing PRKCA, the protective effects of EGCG were significantly attenuated, indicating that PRKCA participates in EGCG protection against LPS-induced lung injury.

Alveolar macrophages are the main resident phagocytes in lung, which participate in the host’s initial defense response through producing inflammatory mediators and chemokines, and regulate the initiation and development of pulmonary inflammation^51,52^. Meanwhile, excessive activation of macrophages is also the key mechanism of inflammatory damage. TNF-α and IL-1β are the key components of cytokine network and the important inflammation mediators of ALI initiation, which mainly secreted by mononuclear macrophages^53,54^. To further address the molecular mechanism underlying PRKCA-mediated EGCG protection against LPS-mediated ALI, we examined the specific effects of PRKCA and the dynamic effects of EGCG and PRKCA on LPS-stimulated mouse macrophage RAW264.7 cells. Consistent with previous studies, within LPS-stimulated RAW264.7 cells, EGCG treatment increased PRKCA protein and reduced proinflammatory cytokine levels; after silencing PRKCA within LPS-induced RAW264.7 cells, EGCG inhibition upon proinflammatory cytokines showed to be significantly attenuated. In summary, PRKCA participates in EGCG protection against LPS-stimulated pulmonary damage through affecting proinflammatory cytokine release by macrophages.

Taken together, the present study revealed that EGCG pretreatment inhibits LPS-induced ALI in mice. The protective mechanism might be associated with the inhibitory effects of PRKCA on proinflammatory cytokine release via macrophages.

## Supplementary information

Supplementary Figures.

## Data Availability

Please contact the authors for data requests.
