# Supplementary material for: EGCG promotes PRKCA expression to alleviate LPS-induced acute lung injury and inflammatory response
Source: Sci Rep. 2021 May 26;11:11014. doi: 10.1038/s41598-021-90398-x (PMC8154949; doi:10.1038/s41598-021-90398-x)
Supplement: Supplementary file 1 — Supplementary Figures. [file 41598_2021_90398_MOESM1_ESM.docx]

**EGCG promotes PRKCA expression to alleviate LPS-induced acute lung injury and inflammatory response**

Mian Wang^1#^, Hua Zhong^2#^, Xian Zhang^1^, Xin Huang^3^, Jing Wang^1^, Zihao Li^1^, Mengshi Chen^1,4*^, Zhenghui Xiao^5^

1 Department of Epidemiology and Health Statistics, Xiangya School of Public Health, Central South University, Changsha 410083, China;

2 Department of Cardiology, Xiangya Hospital, Central South University, Changsha 410008, China;

3 Department of Epidemiology and Health Statistics, Hunan Normal University, Changsha 410006, China;

4 Hunan Provincial Key Laboratory of Clinical Epidemiology, Central South University, Changsha 410083, China;

5 Hunan Provincial Key Laboratory of Pediatric Emergency, Hunan Children’s Hospital, Changsha 410006, China.

^#^ Co-first author, MW and ZH contributed equally to this study.

*Corresponding author: Chen Mengshi, PhD, Department of Epidemiology and Health Statistics, Xiangya School of Public Health, Central South University, 110 Xiangya Road, Changsha 410000, Changsha, China.

1. mail: 121444639@qq.com

Telephone: +86-731-84805454

Fax: +86-731-84805454

**Supplementary Figure legend**

**
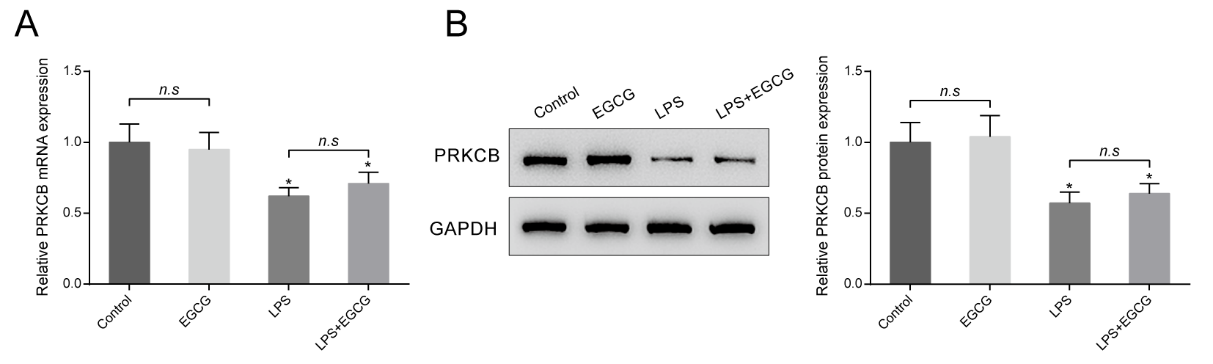
**

**Fig. S1 The effect of EGCG on PRKCB expression in LPS-mediated ALI mice** Mice were divided into four groups (the control group, the EGCG group, the LPS group, and the LPS + EGCG group) accordingly. (A) PRKCB mRNA expression in mouse lung tissues from each group was detected by real-time qPCR. (B) PRKCB protein levels in mouse lung tissues from each group were detected by Immunoblotting. **P*<0.05 compared to Control group.

**High-resolution images of all gels and blots**

Fig.3C-PRKCA





Fig.3C-GAPDH





Fig.4A-PRKCA





Fig.4B-GAPDH





Fig.5B-p-p38





Fig.5B-t-p38





Fig.5B-p-ERK1-2





Fig.5B-ERK1-2





Fig.5B-p-JNK





Fig.5B-JNK





Fig.5B-GAPDH





Fig.6A-PRKCA





Fig.6A-IL-6





Fig.6A-IL-1β





Fig.6A-TNF-α





Fig.6A-GAPDH





Fig.6F-p-p38





Fig.6F-t-p38





Fig.6F-p-ERK1-2





Fig.6F-ERK1-2





Fig.6F-p-JNK





Fig.6F-JNK





Fig.6F-GAPDH





Fig.S1B-PRKCA





Fig.S1B-GAPDH
